# Hypoxia-dependent drivers of melanoma progression

**DOI:** 10.1186/s13046-021-01926-6

**Published:** 2021-05-08

**Authors:** Simona D’Aguanno, Fabiana Mallone, Marco Marenco, Donatella Del Bufalo, Antonietta Moramarco

**Affiliations:** 1 Preclinical Models and New Therapeutic Agents Unit, IRCCS Regina Elena National Cancer Institute, Rome, Italy; 2 Department of Sense Organs, Sapienza University of Rome, Rome, Italy

**Keywords:** Hypoxia, HIF-1, Cutaneous melanoma (CM), Uveal melanoma (UM), Mucosal melanoma (MM), Angiogenesis, Vasculogenic mimicry

## Abstract

Hypoxia, a condition of low oxygen availability, is a hallmark of tumour microenvironment and promotes cancer progression and resistance to therapy. Many studies reported the essential role of hypoxia in regulating invasiveness, angiogenesis, vasculogenic mimicry and response to therapy in melanoma. Melanoma is an aggressive cancer originating from melanocytes located in the skin (cutaneous melanoma), in the uveal tract of the eye (uveal melanoma) or in mucosal membranes (mucosal melanoma). These three subtypes of melanoma represent distinct neoplasms in terms of biology, epidemiology, aetiology, molecular profile and clinical features.

In this review, the latest progress in hypoxia-regulated pathways involved in the development and progression of all melanoma subtypes were discussed. We also summarized current knowledge on preclinical studies with drugs targeting Hypoxia-Inducible Factor-1, angiogenesis or vasculogenic mimicry. Finally, we described available evidence on clinical studies investigating the use of Hypoxia-Inducible Factor-1 inhibitors or antiangiogenic drugs, alone or in combination with other strategies, in metastatic and adjuvant settings of cutaneous, uveal and mucosal melanoma.

Hypoxia-Inducible Factor-independent pathways have been also reported to regulate melanoma progression, but this issue is beyond the scope of this review.

As evident from the numerous studies discussed in this review, the increasing knowledge of hypoxia-regulated pathways in melanoma progression and the promising results obtained from novel antiangiogenic therapies, could offer new perspectives in clinical practice in order to improve survival outcomes of melanoma patients.

## Background

Melanoma originates from melanocytes resident in the skin (cutaneous melanoma, CM), in the choroid, ciliary body, and iris of the eye (uveal melanoma, UM) or in mucosal membranes from different sites (mucosal melanoma, MM). CM, UM and MM are distinct neoplasms in terms of biology, epidemiology, aetiology, molecular characteristic and clinical features. They represent respectively the 91.2, 5.3 and 1.3% of all melanoma cases recorded in the USA [1–3]. Due to its rarity, MM is the least studied subtype among the three.

Reduced levels of oxygen (hypoxia) play a relevant role in melanocyte transformation [4] and represent a common feature in malignant tumor growth, including the growth of CM, UM and MM. Oxygen delivery is compromised within the tumor microenvironment due to abnormal vessels and resulting leaky and slow blood flow. In addition, oxygen consumption is elevated because of the demands of proliferating tumor cells [5, 6]. Clinically, hypoxia in the tumor mass is related to metastasis, chemotherapy and radiotherapy failure, and is indicative of poor prognosis for cancer patients [4, 7, 8]*.* Specifically*,* hypoxia affects tumor aggressiveness through the ability to activate signalling pathways involved in invasiveness, angiogenesis, and epithelial-mesenchymal transition (EMT), an essential mechanism for tumor metastasization [9]. Hypoxia also selects resistant populations of tumor cells, with consequent adaptive response to hypoxic microenvironment [10, 11]*.*

The adaptive response to hypoxia is driven by several transcription factors, including hypoxia-inducible factor 1 (HIF-1), the key oxygen sensor and principal regulator of hypoxia-mediated gene response, along with HIF-2 [12], cyclic AMP response-element binding protein (CREB) [13]*,* and AP-1 [14]*.* HIF complex is composed of an alpha subunit (HIF-1α, 2α or 3α) regulated by oxygen and a stable 1 beta subunit (HIF-1β) constitutively expressed [15].

**Fig. 1:**
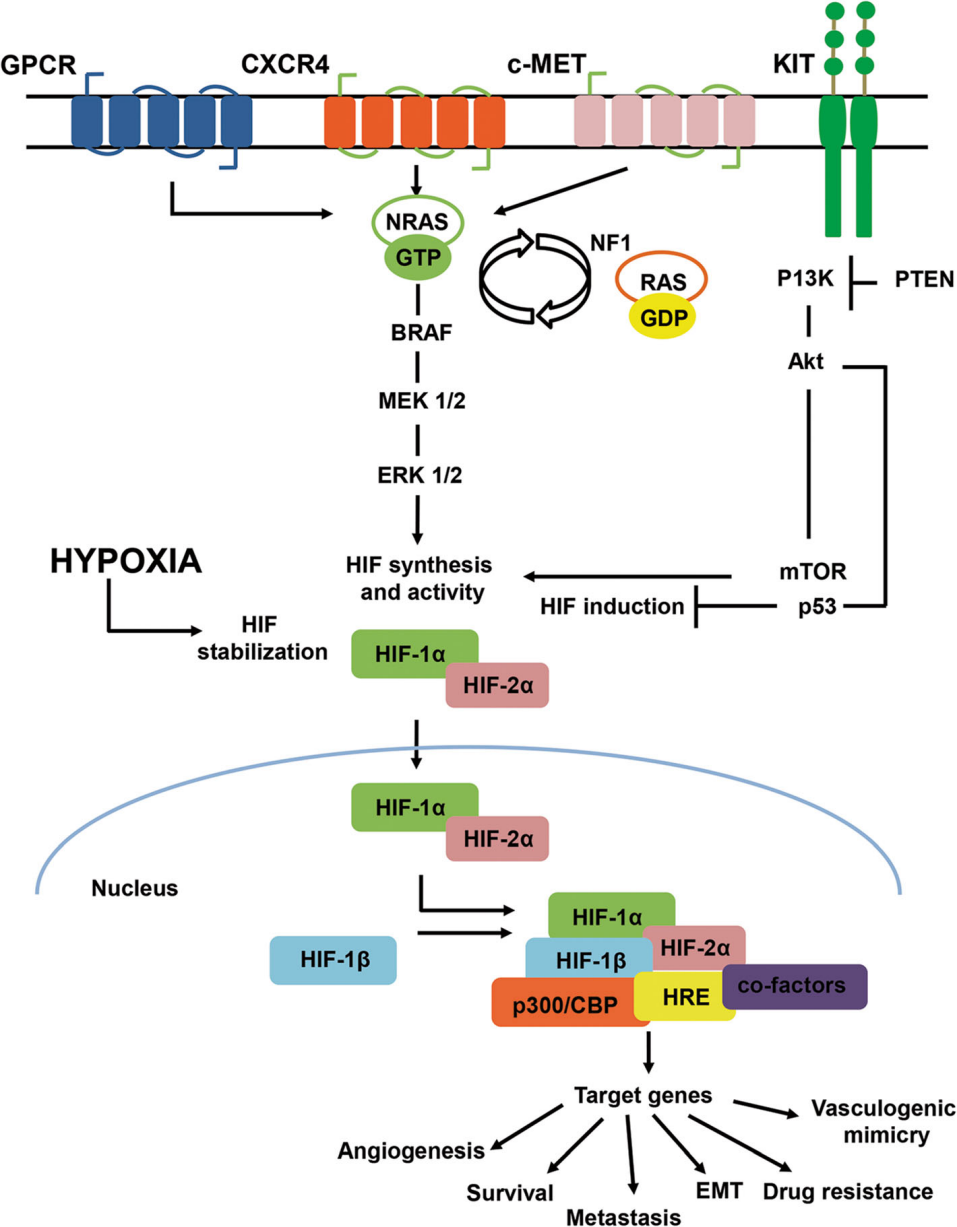
Signalling pathways involved in CM, UM and MM oncogenesis, and regulation of HIF under hypoxia. The G protein-coupled receptor (GPCR) and its Gα subunits GNAQ and GNA11, downstream activate MEK1/2. The C-X-C chemokine receptor 4 (CXCR4) and c-Met activate the mitogen-activated protein kinase (MAPK) signalling pathway, consisting of BRAF-MEK1/2-ERK1/2. The KIT receptor activates the PI3K/Akt/mTOR pathway, which is influenced by phosphatase and tensin homolog (PTEN), inhibiting  p53. Loss of NF1 in melanoma promotes transition of RAS/GDP (inactive state) to its active state NRAS/GTP, in turn activating the BRAF-MEK1/2-ERK1/2 and PI3K/Akt/mTOR pathways. All of these pathways promote HIF-1α and HIF-2α synthesis and activity. Hypoxia drives HIF-1α and HIF-2α stabilization into the nucleus, with consequent induction of several genes involved in angiogenesis, survival, metastasization, EMT, drug resistance, vasculogenic mimicry through p300/CBP binding to HRE

Hypoxia drives stabilization and translocation to the nucleus of HIF-1α and HIF-2α with consequent interaction with the transcriptional coactivators CREB-binding protein (CBP) and p300 that bind to hypoxia-responsive elements (HRE) in the promoter region of more than 300 target genes (Fig. 1) [16, 17]. HIF-1α and HIF-2α share similar domain structure and bind to the same DNA sequence, however, they differ in their transactivation domains resulting in distinct transcriptional cofactors and target genes, with tissue-specific expression patterns [12, 18]*.* On the contrary, the relevance of HIF-3α for the regulation of the HIF pathway is not completely understood, although it is usually regarded as a negative regulator of HIF-1α and HIF-2α [19, 20].

**Fig. 2:**
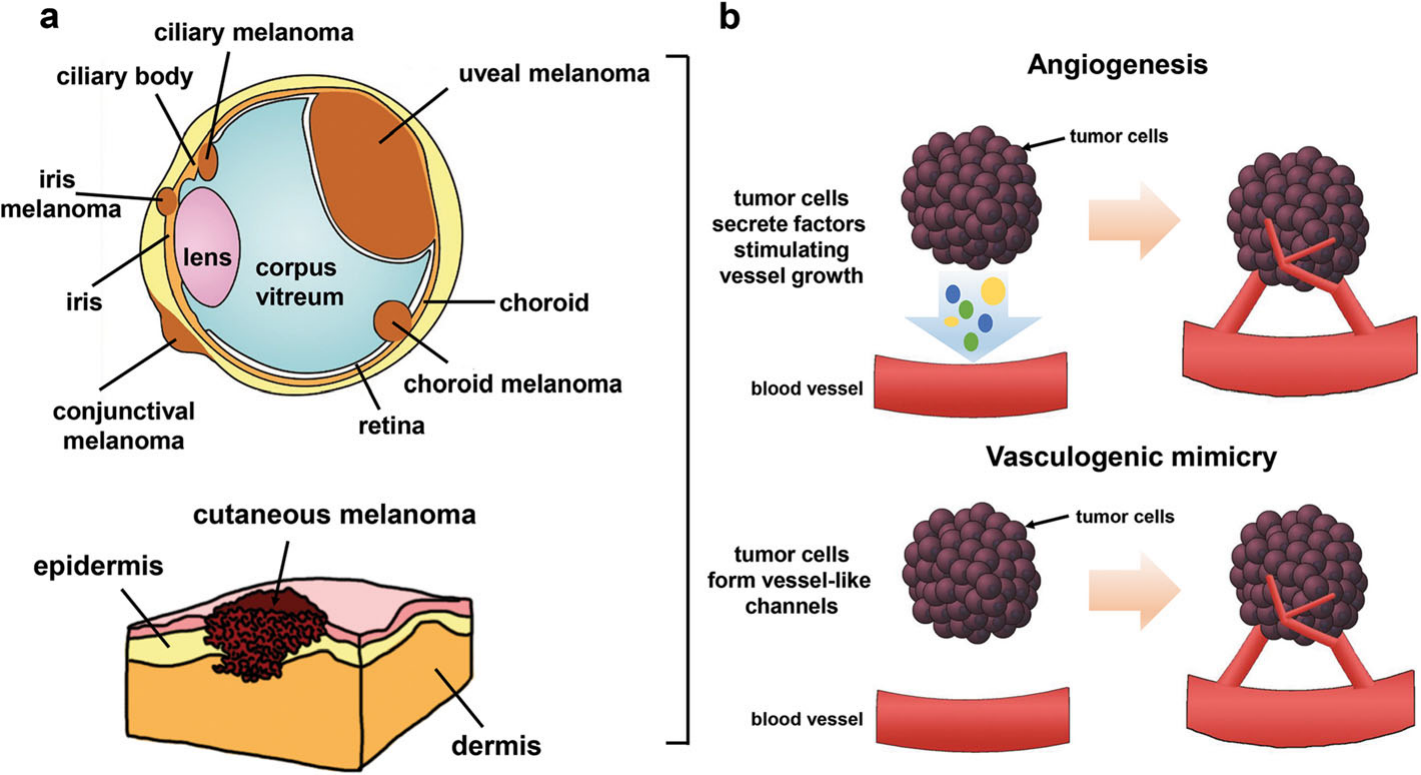
**a** Graphical representation of different melanoma types.** b** Different mechanisms underlying angiogenesis and vasculogenic mimicry in CM, UM and MM. In angiogenesis, cancer cells secrete angiogenic factors and promote the development of new endothelial cell-lined blood vessels from pre-existing normal blood vessels. Conversely, in vasculogenic mimicry tumor cells mimic the presence and function of endothelial cells, forming capillary-like structures

Multiple evidence also demonstrated oxygen-independent regulation of HIF signaling. This phenomenon has been indicated by several authors as “pseudohypoxia” and has been reported to be induced by genetic alterations in genes including VHL, MDM2, TP53, Rack, HSP90, metabolites as succinate, fumarate and 2-hydroxyglutarate, or noncoding RNA [21–23]. In this review we report on hypoxia-dependent drivers of progression of different melanoma subtypes (Fig 2a). From a survey in PubMed (https://pubmed.ncbi.nlm.nih.gov/) a list of papers with the “melanoma” and” hypoxia” or “HIF” were retrieved and analysed focusing our attention on those published in the last 20 years.

## Cutaneous melanoma 

### Epidemiology, diagnosis and therapy

The worldwide incidence of CM has dramatically increased in the recent years [24, 25]. It differs significantly between men and women: while young women are more susceptible to the disease, after the age of 40, men are more affected than women. CM is more common in fair-skinned Caucasian populations with a peak of presentation at the seventh and eighth decades of life. Every year, more than 140,000 new cases of melanoma are diagnosed in Europe, while the American Cancer Society estimates approximately 106,110 new melanoma diagnoses in the United States for 2021 [26, 27]. The main environmental risk factor for CM development is represented by ultraviolet light radiation from sunlight exposure [28] and, in particular, a history of sunburn in childhood or adolescence [29]. Congenital and acquired melanocytic nevi, genetic susceptibility and family history have been reported as additional predisposing factors [30, 31]. Moreover, somatic genetic alterations have been found to be associated with CM [32]. The BRAF/RAS/RAF/MEK/ERK signalling pathway is frequently altered in CM, with 40–50% of CM presenting BRAF mutations, 20–30% NRAS mutations, 10–15% NF1 mutations and 1–3% KIT mutations [32]. Approximately 80–90% of BRAF mutations are V600E (valine to glutamic acid), while 5–12% are V600K (valine to lysine) and ≤ 5% are V600D (valine to aspartic acid) or V600R (valine to arginine) [24, 32–34]. CM is characterized by early lymphatic dissemination in the regional lymph nodes and by lymph node metastases that significantly influence the staging, prognosis and clinical approach [35]*.* Lymphangiogenesis in CM is induced and maintained by growth factors, amongst which the most efficient are the VEGF-C and VEGF-D that bind to their specific vascular endothelial growth factor receptor, VEGFR-3, expressed by lymphatic endothelial cells [35].

CM is categorized by Tumor-Node-Metastasis (TNM) staging to define patients with local disease (stage I-II), node-positive disease (stage III), and advanced or metastatic disease (stage IV). Most CM are localized at the time of initial clinical presentation and are successfully treated with surgical excision with adequate margins [25].

The most common sites of CM metastases are distant skin and lymph nodes followed by lung, liver, brain and other areas of the body. 5-year survival rates range from 98 to 20% passing from localized to advanced metastatic melanoma.

Great progress has been made in recent years in the treatment of patients with unresectable or metastatic melanoma, with the development of MAPK molecular targeted therapy directed at oncogenic BRAF (vemurafenib and dabrafenib) alone or in combination with MEK inhibitor (cobimetinib and trametinib). Good results were also achieved with the development of immune checkpoint blockade strategies targeting the programmed death receptor-1 (PD-1) or the cytotoxic T-lymphocyte–associated antigen 4 (CTLA-4) [25].

### HIF-regulated pathways

#### HIF-1 expression and regulation

Similarly to that observed in 50–60% of advanced solid tumors, CM is characterized by areas of hypoxia/anoxia that result from an imbalance between oxygen supply and consumption in proliferating tumor cells [36]. Studies have shown that the presence of hypoxia within a tumor is an independent marker of a poor prognosis for patients with various cancer types, including CM [37].

Constitutive low level of tissue oxygenation is a peculiar characteristic of rodent and human epidermis due to the absence of vasculature. As a consequence, both rodent and human epidermis show increased levels of HIF-1α in the basal epidermal compartment [38, 39].

Melanocytes, which are located along the basement membrane in the basal layer of the epidermis in humans and mostly in hair follicles in mice, show elevated/stabilized HIF-1α, that drives their growth and increases survival under hypoxic conditions [36]. In some cases, melanocytes showed an uncontrolled proliferation only when exposed to hypoxia: this is the case of melanocytes expressing KIT mutation [34].

Microphthalmia-associated transcription factor (MITF), which plays a relevant role in the regulation of normal melanocyte development, is responsible for pigment cell-specific transcription of the melanogenesis enzyme genes [40] and elicits oncogenic melanocyte transformation [41, 42]. MITF is reported to bind directly to the HIF-1α promoter, favouring melanocytes survival under normoxic conditions [43]. On the other hand, MITF expression in primary melanocytes and melanoma cells is suppressed under hypoxic conditions by the HIF-1α mediated activation of a transcriptional repressor, the differentially expressed in chondrocytes protein-1. This leads to melanoma cell growth arrest, as reported in vitro and in vivo experiments [44], thus suggesting MITF-targeted therapeutic approaches in melanoma.

BRAFV600E mutation was found to directly regulate HIF-1α expression in CM [45]. Increased HIF-1α promoted by BRAFV600E could contribute to intensify and foster melanoma genesis in association to PI3K-Akt pathway, which is known to play a relevant role in early melanoma [46]. In line with these evidences, decreased HIF-1α expression was observed after pharmacologic inhibition of BRAF by using sorafenib, a dual inhibitor targeting RAF/MEK/ERK pathway in CM cells, and tyrosine kinase VEGFR and platelet-derived growth factor receptor (PDGFR) in tumor vessels [45]. Bedogni et al showed that Notch1 is highly expressed in melanoma and represents a link between the Akt as well as hypoxia/HIF-1α pathways [36]. Moreover, Akt and hypoxia additively regulate Notch1 transcriptionally in melanocytes [47].

We previously reported the ability of the antiapoptotic protein Bcl-2to increase HIF-1α protein stability in CM cells under hypoxia through a mechanism involving the molecular chaperone HSP90 [48].

Constitutive expression of HIF-1α has been detected by immunohistochemical analysis in CM sections, quantitative PCR, western blotting and immunonofluorescent staining of CM cells [49–51]. Higher expression of HIF-1α in CM cells than in benign nevi has been evidenced by immunohistochemistry, with HIF-1α detection in both nucleus and cytoplasm [52]. Marconi and colleagues analysed the expression of HIF-1α in 12 skin samples derived from patients with different CM subgroups, demonstrating a correlation between HIF-1α expression and degree of malignancy. In detail, HIF-1α was found to be scarcely expressed in the less aggressive types of CM, whereas it was highly represented in CM invading the dermis [53]. These results, limited by the sample size, were confirmed in a more recent study including 376 patients diagnosed with CM from 29 participating centres. Specifically, HIF-1α expression, although identified in a small percentage of CM, was associated with the most aggressive characteristics [54].

#### Angiogenesis

As several authors have extensively revised the role of angiogenesis in melanoma, this issue has not been deeply discussed in this section [55–57].

One of the most important target genes of HIF-1 is VEGF. VEGF-A, commonly referred to as VEGF, can be processed by alternative gene splicing into at least five isoforms, including isoforms of 121, 143, 165, 189 and 206 amino acid length [58]. In human melanoma xenograft models, overexpression of VEGF121 and VEGF165 isoforms resulted in aggressive tumor growth, whereas cells overexpressing VEGF189 remained non-tumorigenic and dormant [59]. VEGF is involved in the autocrine and paracrine regulation of CM progression and, in particular, VEGF-A expression has been correlated with the transition of melanoma lesions from radial growth phase, characteristic of primary tumor, to a vertical growth phase, indicative of melanoma progression [60]. VEGF-A exerts its biological effects on endothelial cells through the interaction with VEGFR-1 and VEGFR-2. VEGFR-1 mainly mediates endothelial cell migration in response to the angiogenic factor, while VEGFR-2 is the major receptor involved in endothelial cell survival and proliferation and in the increase of microvascular permeability [61]. In a study involving 183 CM patients, divided into four subgroups according to the Breslow score, and 139 healthy individuals, the serum level of VEGF was found higher in CM patients, suggesting its prognostic value [62].

VEGF expression was also evaluated in a cohort of 376 patients, demonstrating frequently elevated expression in CM but no relationship with tumor aggressiveness [50].

We previously evidenced that Bcl-2 overexpression in human CM models enhanced hypoxia-induced VEGF mRNA stability and promoter activation, thus promoting angiogenesis [63, 64]. More recently also the overexpression of the antiapoptotic protein, Bcl-xL, has been reported to induce VEGF secretion, HIF-1α protein expression and angiogenesis in CM [65].

#### Vasculogenic mimicry

Hypoxia also regulates the vasculogenic mimicry,  a process firstly identified in CM and UM in vitro and in vivo*.* It represents a mechanism through which tumor cells mimic the presence and function of endothelial cells, forming capillary-like structures that provide blood supply to the tumor (Fig. 2 b) [66, 67]. Findings regarding the ability of vasculogenic mimicry to supply nutrients and oxygen in the early stage of melanoma growth have been also reported, as well as results demonstrating the role of angiogenesis to replace vasculogenic mimicry for blood supply in late stage of melanoma growth [68]. A meta-analysis study across 15 cancer types, including CM, revealed that tumor vasculogenic mimicry is associated with poor prognosis [69]. Vasculogenic mimicry during hypoxia has been reported as a possible mechanism to explain the limited efficacy of antiangiogenic therapies [70].

Interestingly, the tumor hypoxic region characterized by linearly patterned programmed cell necrosis, with cells positive for caspase-3, caspase-9 and Bax and negative for TUNEL staining, has been suggested to provide a spatial foundation for vasculogenic mimicry [71]. Moreover, melanoma xenografts obtained inoculating mouse melanoma cells into mouse ischemic limbs, showed an increase in vasculogenic mimicry under ischemic condition, and higher expression of HIF-1α, VEGF, matrix metalloproteinases 2 (MMP2), and MMP9 [72]. Silencing of HIF-1α and HIF-2α was found to completely inhibit melanoma capillary-like structure formation, indicating that vasculogenic mimicry in melanoma cells is controlled by HIF-dependent transcriptional mechanism [73]. Hypoxia-related HIF-1α stabilization has been also reported to enhance expression/activation of the Met proto-oncogene in melanoma cells, which in turn promotes formation of capillary-like structures by vasculogenic mimicry [74].

While investigating the presence of bidirectional autocrine/paracrine endothelin-1 (ET-1)/endothelin-1 receptor signalling in melanoma cells, blood and lymphatic endothelial cells, hypoxia was observed to enhance ET-1 expression regulating vascularization and cell motility through increased secretion of VEGF-A and VEGF-C in response to HIF-1α and HIF-2α [73].

#### Invasion

HIF-1α was also reported to be involved in the regulation of tumor progression-associated properties. Specifically, it induced EMT, by regulating the transcription factor TWIST through the HRE located in the TWIST proximal promoter [75, 76]. In accordance, altered TWIST expression was found to be correlated with shortened survival in patients with CM [77].

Through HIF-1α, hypoxia has been also reported to contribute to CM heterogeneity, and to trigger metastatic progression by driving a switch from a proliferative to an invasive phenotype [78].

Analysing the gene expression profile of a set of human CM cell lines, Widmer and colleagues found that exposure of proliferative melanoma cells to hypoxic microenvironments was sufficient to downregulate melanocytic marker expression and increase their invasive potential in a HIF-1α dependent manner [78].

Hypoxia, through HIF-1, increased tumor cells invasion by directly regulating genes that are involved in the degradation and remodelling of the extracellular matrix (ECM), including urokinase-type plasminogen activator receptor (uPAR), MMP2 and protease-activated receptor 1 [79–81]. The uPA/uPAR system components are currently recognized as important prognostic and predictive markers of malignancy [82]. A recent paper reported high uPAR expression and vascular proliferation index as novel markers of reduced cancer specific survival in primary melanoma, and confirmed an association between uPAR and angiogenesis [83]. It was reported that hypoxia promotes spontaneous lymph node metastasis in human melanoma xenografts by up-regulating uPAR [84]. Both increased uPAR mRNA stability and expression, due to the presence of HRE at the uPAR promoter, have been reported under hypoxia in different tumor histotypes, including CM [85–87]. We previously reported that overexpression of Bcl-2 in CM cells exposed to hypoxia was able to modulate uPAR expression through Sp1 transcription factor [87].

In 2014, a pathway able to promote the MMP2 activation induced by hypoxia has been reported through the involvement of CD147, an extracellular matrix metalloproteinase inducer particularly expressed in melanoma [88, 89]. Chromosome immunoprecipitation assay, performed in CM cell lines under hypoxia, demonstrated that HIF-1α directly binds to CD147 promoter, causing MMP2 activation [88].

We demonstrated that Bcl-2 and Bcl-xL proteins cooperated with hypoxia to induce the expression of several metalloproteases, including MMP2, in melanoma models [65, 90].

HIF-1α and HIF-2α proteins were found to drive CM invasion and invadopodia formation through PDGFRα and focal adhesion kinase (FAK) mediated activation of proto-oncogene SRC and by coordinating ECM degradation via membrane type 1 (MT1)-MMP and MMP2 expression. Evidences were also provided about the ability of HIF-1α and HIF-2α to independently activate SRC to promote CM metastases [91].

Mass spectrometry analysis of the HIF-2α nuclear interactome in melanoma cells revealed that master proteins involved in melanoma development, such as MITF and SOX10, are HIF-2α binding proteins [92]. HIF-2α protein was also found to increase cell migration and drug resistance in CM by directly regulating the transcriptional factor Snail [93], and to mediate stemness of melanoma cells by regulating the microRNA-363-3p/p21 axis [94]. Intriguingly, selective stabilization of HIF-2α was found to reduce tumor growth and angiogenesis by promoting secretion of a soluble form of the VEGF receptor (sVEGFR-1) from tumor-associated macrophages, which can inhibit VEGF biological activity, as evaluated in a murine melanoma model [95].

Several findings also reported the ability of hypoxia to trigger the expression of both carbonic anhydrase IX (CAIX) and XII (CAXII), two cell-surface metalloenzymes involved in the regulation of intra- and extracellular pH, cell adhesion and migration, as well as survival of melanoma patients [96]*.* The levels of HIF-1α and CAIX proteins under hypoxia were decreased in melanoma cells exposed to vemurafenib in a cell type-dependent manner, thus indicating hypoxia involvement in the response to drug treatment and in the induction of heterogeneity [97]*.* A recent paper also evidenced CAXII expression in melanoma cell lines and the role of Hedgehog pathway as mediator of melanoma cell migration and CAXII expression under normoxic, as well as hypoxic microenvironment [98]*.*

#### Autophagy and other pathways

Experimental evidence for the role of HIF-1α in hypoxia-regulated microRNA expression have also been reported [99]. MiR-210, a direct target of HIF-1α [99], was found to be significantly higher in metastatic CM biopsies compared to primary ones, and circulating miR-210 in plasma allowed to identify early systemic metastasis recurrence in melanoma patients [100]. On the other hand, miR-199a-5p, miR-18b, miR-138 and miR-33a/b have been reported to inhibit proliferation, tumor growth, and invasion in melanoma by targeting HIF-1α [101–104].

Together with other pathways induced by hypoxia through the HIF-signalling, also glycolysis contributes to CM progression [36]. HIF-1α is known to be a master regulator of glycolysis though the direct regulation of expression of glycolysis enzymes, such as glucose transporters 1/4 (GLUT 1/4), hexokinase 2, pyruvate kinase M2 and lactate dehydrogenase A (LDHA) [105]. HIF-1α protein upregulation and stabilization lead to glycolysis induction in CM cells, and to CM development and progression both in the presence and in the absence of oxygen [45, 49]. The HIF-1α target gene GLUT1, was found to be elevated in metastatic and BRAFV600E mutated melanoma cell lines under hypoxic conditions [106].

Hypoxia is among the stress factors able to induce autophagy. Different studies reported evidences of an existing link between hypoxia, autophagy, and tumor progression in CM [107]. Hypoxia was able to increase the proangiogenic behaviour of murine melanoma deficient for beclin1 [108]. Blocking hypoxia-induced autophagy in murine melanoma models was able to restore the activity of cytotoxic T-cells and to induce tumor regression [109]. Moreover, hypoxia was reported to reduce the expression of the antimetastatic pigment epithelium-derived factor through autophagy-dependent and HIF-1-independent manner [110]. Analysing 79 CM, Sivridis et al. observed a correlation between expression of the two autophagy-related proteins Beclin 1 and light chain 3A (LC3A), and HIF-1α immunoreactivity [111].

A huge amount of data evidenced the involvement of hypoxia in the regulation of survival, apoptosis and response to therapy in CM. A detailed discussion of the contribution of hypoxia to these pathways is beyond the scope of this review as it was reviewed elsewhere by other authors [112–115]*.*

### Preclinical studies with drugs targeting HIF-1, angiogenesis or vasculogenic mimicry

Hypoxia has been found to promote drug resistance in melanoma. Current therapeutic strategies employing the use of BRAF or MEK inhibitors do not completely avoid the onset of resistance phenomena in patients. Qin and colleagues, by using melanoma 3D spheroids and 2D hypoxic cultures, demonstrated that hypoxia-driven upregulation of hepatocyte growth factor (HGF)/MET pathway plays an important role in vemurafenib resistance [116].

Hypoxia has been also reported to induce CM metastasis and drug resistance through HIF-2α-dependent activation of Snail1 and reduced expression of E-cadherin [93]. Performing chromosome immunoprecipitation and luciferase reporter assays, the direct binding of HIF-1α to HRE element in the PD-L1 proximal promoter was demonstrated in melanoma cells. As a consequence, under hypoxia a rapid, dramatic, and selective up-regulation of PD-L1 on splenic myeloid-derived suppressor cells was observed in melanoma-bearing mice model [117].

#### HIF inhibitors

Inhibition of HIF-1α has been reported to reduce metastasization of cancers from different origin, such as lung and breast carcinoma [91, 118]. HIF-1α also represents an attractive therapeutic molecular target in CM. A transferrin-polyethylenimine-HIF-1α–short-hairpin RNA complex was used to target HIF-1α obtaining a dramatic inhibition of tumor growth in melanoma xenograft model [119].

Drug targeting HIF-1α protein stability could also modulate glycolysis. Treatment of mouse melanoma model with isoliquiritigenin, a natural flavonoid compound, caused reduced stability of HIF-1α, responsible for a reduced expression of its target glycolysis enzymes, GLUT 1/4, hexokinase 2, pyruvate kinase M2 and LDHA, finally resulting in induction of apoptosis [105, 120].

A broad spectrum of molecules of natural origin with direct in vitro and in vivo pharmacological effects on migration and/or metastasis of melanoma cells have been evaluated, among which also molecules affecting HIF-1α expression [121]. Luteolin, a natural flavonoid, found in fruits, vegetables and medicinal herbs, was reported to reduce EMT, angiogenesis and metastasis in CM through downregulation of HIF-1α, p-Akt, VEGF-A, p-VEGFR-2, MMP2, and MMP9 proteins expression, although the pharmacological action and mechanism of luteolin on the metastasis of melanoma was not completely elucidated [122]. Also vanillin has been found to inhibit cell migration by repressing STAT3-mediated HIF-1α mRNA expression in CM cells [123].

#### Antiangiogenic drugs

Antiangiogenic therapies approved for the treatment of a variety of solid tumors included the monoclonal antibody (mAb) bevacizumab (Avastin) targeting VEGF-A, aflibercept, the VEGF TRAP recombinant chimeric soluble receptor capable of VEGF-A, VEGF-B and placental growth factor (PIGF) inhibition, and the mAb ramucirumab targeting VEGFR-2. Being already evaluated or under evaluation in clinical trials, preclinical studies using these drugs will not be discussed in this section.

Biological effects of other VEGFR-1 antagonistic peptides or peptide mimetics and mAb have been demonstrated in preclinical studies for different solid tumors, including melanoma [124]. In particular, a marked reduction of tumor volume was observed in a syngeneic murine model of melanoma after treatment with the anti-human VEGFR-1 mAb D16F7, that recognizes also the murine and rat protein [125]. Inhibition of angiogenesis, tumor infiltration by myeloid cells, myeloid progenitors mobilization, tumor cell chemotaxis, ECM, bone marrow invasion and endothelial cell migration have been also observed after exposure to D16F7 [124]. In vivo treatment of CM murine syngeneic model with D16F7 also inhibited tumor growth and increased the activity of immune checkpoint inhibitors [126].

Starting from the structure of sorafenib, Sun and colleagues designed and synthesized a series of molecules acting as VEGFR-2 and Epidermal Growth Factor Receptor (EGFR) dual inhibitors [127]. Among them, two compounds showed stronger inhibitory activities against both EGFR and VEGFR-2, and induced in vitro antiproliferative effect in human and murine melanoma cells, as well as in vivo antitumor activity in melanoma xenograft models [127].

An inhibitor of the FAK scaffold has been reported to inhibit in vitro cell proliferation of a panel of human melanoma cell lines, and to reduce angiogenesis and lymphatic vessel density in melanoma xenograft tumors. This effect was due to the disruption of FAK-VEGFR-3 complex [128].

The treatment of human CM cell lines with the herbal medicinal mushroom, Cordyceps militaris, resulted in reduced VEGF secretion by tumor cells and apoptosis induction associated with decreased Akt1 and GSK-3β activation [129]. Treatment of CM xenograft with Cordyceps militaris also caused a significant reduction of tumor growth and inhibition of angiogenesis associated to reduced VEGF expression [129].

Also the administration of 16-kDa N-terminal fragment of prolactin, complexed with liposomes, inhibited tumor growth and angiogenesis in mouse CM model, although the mechanism of action has not been elucidated [130].

The inhibition of vasculogenic mimicry may represent an additional valid strategy to fight CM progression. To this purpose, a series of drugs have been tested in CM models. Very recently a protein kinase C inhibitor anchored BRD4 PROTAC loaded PEGylated nanoliposomes was used to inhibit angiogenesis using human umbilical vein endothelial cells-based matrigel basement membrane model. In addition to angiogenesis, this compound inhibited vasculogenic mimicry, cell migration, as well as colony formation in vemurafenib-resistant CM cell lines [131]. Also VEGFR-1 blocking has been found to induce vasculogenic mimicry inhibition [124].

Vasculogenic mimicry has been also associated to CM resistance to dacarbazine (DTIC). In a recent paper, lupeol has been demonstrated to be a proficient agent in treating CM, inhibiting vasculogenic mimicry, thus playing a possible role in overcoming DTIC-induced drug resistance [132].

Apatinib, a small-molecule tyrosine kinase inhibitor, has been reported to decrease the expression of VEGFR-2, and downregulate the ERK1/2/PI3K/MMP2 signalling cascade, thus inhibiting angiogenesis and the development of vasculogenic mimicry in models of CM [133].

The copper (II) complex CPT8 reduced vascular channel formation of aggressive mouse melanoma cells through regulation of reactive oxygen species production and the expression of MMP2 [134].

Nicotinamide, tested in primary CM cultures, effectively inhibited the formation of vasculogenic mimicry structures and destroyed already formed ones, in a dose-dependent manner, through downregulation of VE-Cadherin [135].

Also lycorine hydrochloride has been reported to reduce vasculogenic mimicry by decreasing VE-cadherin gene expression and diminishing its exposure at the cell surface [136].

Targeting of melanoma vascular mimicry using a phenyl-quinoline derivative (CVM-1118) has been evidenced by Hendrix group with no description of whether UM or CM cells were used for the study. Besides the reduction of number of junctions and tubules, as well as of the total tubule length, the compound also showed antiproliferative and proapoptotic activity in melanoma cells. CMV-1118 also acted through inhibition of both stem cell-associated genes and the vascular signalling associated gene VEGF-A. Surprisingly, phosphorylation and consequent stabilization of HIF-1α protein has been also observed after treatment with the phenyl-quinoline derivative [137].

Table 1 reports a list of preclinical studies with HIF inhibitors and antiangiogenic drugs in CM.

**Table 1:** Preclinical studies with drugs targeting HIF-1, angiogenesis or vasculogenic mimicry in cutaneous melanoma

Drug	Involved Pathways	References
Transferrin-polyethylenimine-HIF-1α–short-hairpin RNA complex	Prevention of cell proliferation and induction of cell apoptosis; Downregulation of HIF-1α	Liu Y et al Mol Ther 2009 [119]
Isoliquiritigenin	Reduced expression of HIF-1α and glycolysis enzymes (GLUT 1/4, hexokinase 2, pyruvate kinase M2 and LDHA)	Wang Y et al Recent Pat Anticancer Drug Discov 2016 [120]
Luteolin	Reduced EMT, angiogenesis and metastasis through downregulation of HIF-1α, p-Akt, VEGF-A, p-VEGFR-2, MMP2, and MMP9	Li C et al Phytother Res 2019 [122]
Vanillin	Inhibition of cell migration by repressing STAT3-mediated HIF-1α mRNA expression	Park EJ et al Int J Mol Sci 2017 [123]
Anti-human VEGFR-1 monoclonal antibodies, D16F7	Inhibition of angiogenesis and endothelial cell migration in vitro; in vivo increased activity of immune checkpoint inhibitors	Lacal PM et al Pharmacol Res 2018 [124] Lacal PM et al Pharmacol Res 2020 [126]
Sorafenib analogs	In vitro antiproliferative effect; in vivo anti-tumor activity; inhibitory activities against both EGFR and VEGFR-2	Sun S et al Molecules 2017 [127]
FAK inhibitor	In vitro inhibition of cell proliferation; in vivo reduced angiogenesis and lymphatic vessel density through disruption of FAK-VEGFR-3 complex	Kurenova E et al Cell Cycle 2014 [128]
Cordyceps militaris extract	Apoptosis induction, reduced VEGF secretion, decreased Akt1 and GSK-3β activation	Ruma IMW et al Int J Oncol 2014 [129]
16-kDa N-terminal fragment of prolactin complexed with liposomes	Reduced in vivo tumor growth and angiogenesis	Kinet V et al Cancer Lett 2009 [130]
Kinase C inhibitor	Inhibition of angiogenesis, vasculogenic mimicry, cell migration and colony formation	Fu Y et al Exp Cell Res 2020 [131]
Lupeol	Inhibition of vasculogenic mimicry	Bhattacharyya S et al Microvasc Res 2019 [132]
Apatinib	Inhibition of angiogenesis and vasculogenic mimicry by decreased expression of VEGFR-2, downregulation of the ERK1/2/PI3K/MMP2 signalling cascade	Liu ZJL et al PLoS One 2018 [133]
Copper (II) complex CPT8	Reduced vasculogenic mimicry through downregulation of MMP2	Shi X et al Dalton Trans 2018 [134]
Nicotinamide	Inhibition of vasculogenic mimicry through downregulation of VE-Cadherin	Itzhaki O et al PLoS One 2013 [135]
Lycorine hydrochloride	Reduced vasculogenic mimicry by decreasing VE-cadherin	Liu F et al Pigment Cell Melanoma Res 2012
Phenyl-quinoline derivative (CVM-1118)^a^	Inhibition of vasculogenic mimicry, antiproliferative and proapoptotic activity; inhibition of stem cell-associated genes and VEGF-A gene	Hendrix MJC et al Pharmacol Ther 2016 [137]

^a^In this article the use of Cutaneous Melanoma or Uveal Melanoma cells is not specified

### Clinical trials with antiangiogenic drugs

The effect of angiogenesis inhibition on CM has been investigated in clinical trials [138]. While most studies are based on inhibition of VEGF signalling, others focus on evaluating the effect of multikinase inhibitors of VEGFR − 1, − 2 and − 3, PDGFR -α and β, or KIT.

Inhibition of angiogenesis by using bevacizumab has achieved little impact on CM patient survival [139, 140]. In the AVAST-M trial patients with resected stage IIB, IIC and III CM that received adjuvant bevacizumab showed improved disease-free interval but not overall survival (OS) [141]. Nevertheless, in a recent study, Fane and colleagues, based on the observation that VEGF was expressed at higher level in older CM patients, re-analysed data from the AVAST-M trial finding an age-related difference in response to bevacizumab [139]. Although VEGF was decreased during aging, thereby reducing response to bevacizumab, angiogenesis was increased because of an increase of Secreted Frizzled Related Protein 2 (sFRP2), a modulator of Wnt signalling, in the aged tumor microenvironment [139]. A phase 2 trial of bevacizumab and high-dose interferon alpha-2b (IFN-α2b) was performed in metastatic CM, demonstrating 24% RECIST (Response Evaluation Criteria in Solid Tumors) response and 20% stabilization of disease [142]. The combination of bevacizumab, carboplatin, and paclitaxel ± everolimus was studied in a phase II trial (Alliance). This study resulted in clinical benefit in both arms, although, the addition of everolimus to the combination, increased toxicity and failed to improve outcomes [143].

The use of ramucirumab with or without DTIC in a phase II randomized study on chemotherapy-naïve patients diagnosed with CM resulted in acceptable safety profile. However, survival outcomes were improved with combination therapy [144].

The safety and clinical effects of pazopanib, a multitargeted tyrosine kinase inhibitor, was evaluated in combination with paclitaxel in chemotherapy-naive patients with metastatic CM, demonstrating significant activity with recorded 37% objective response rate and 55% stable disease, and an overall clinical benefit rate of 93% [145].

The antiangiogenic activity of aflibercept was investigated in a multicenter phase II study on 28 patients with inoperable stage III or IV CM and no prior chemotherapy, showing promising results. The study demonstrated 10% response rate, with 3.7 months mPFS and 16.3 months median OS [146]. In addition, a randomized phase II study evaluating sequential therapy with aflibercept and high-dose IL-2 versus high-dose IL-2 alone for inoperable stage III or IV melanoma, demonstrated superior antitumor efficacy with combination therapy compared to monotherapy [147].

Treatment with thalidomide, an angiogenic and immunomodulatory compound, was evaluated as single agent or as combinational therapy in metastatic CM, demonstrating insufficient activity and safety profile to warrant further investigation [148–150]. Similarly, lenalidomide, a thalidomide analog with immunomodulatory and antiangiogenic properties, was evaluated in advanced CM, resulting in limited clinical benefit [151–153]. In line with preclinical data [154], several phase II trials evaluating the efficacy of imatinib, a PDGFR and KIT inhibitor, in metastatic CM reported poor OS outcomes [155, 156]. However, single-agent activity of imatinib was reported in melanoma patients whose tumors harboured activating KIT mutations, warranting further investigation in this subset of patients [157, 158]. Moreover, nilotinib, a KIT-selective tyrosine kinase inhibitor, was evaluated in the phase II TEAM trial in patients with KIT-mutated advanced melanoma, resulting in similar activity to historical data from imatinib-treated patients [159]. In this direction, the efficacy of sunitinib, a multikinase inhibitor of VEGFR-1-3, PDGFR, and receptor tyrosine kinase KIT, was investigated in a phase II clinical trial for treatment of metastatic CM, demonstrating clinical benefit limited to CM patients harbouring KIT mutations [160].

A multicenter, open-label, phase II study evaluated the safety and clinical activity of axitinib, a selective inhibitor of VEGFR-1, -2, and -3, as single agent in metastatic CM. Results were promising, showing 18.8% objective response rate with a median response duration of 5.9 months [161]. In agreement, results from a prospective phase II clinical trial of axitinib followed by cytotoxic chemotherapy with carboplatin and paclitaxel demonstrated high activity in metastatic CM, reaching an objective response rate of 22% and a disease control rate of 66.7% [162].

The efficacy of recombinant human endostatin, an endogenous angiogenic inhibitor, was tested in a phase II, two-armed clinical trial for the treatment of metastatic CM. Specifically, the combinational treatment with endostatin plus DTIC was compared to DTIC plus placebo, resulting in significant improvement in mPFS and OS in favour of the endostatin plus DTIC combination [163].

Sorafenib was used in combination with DTIC as first-line therapy for advanced melanoma with acceptable toxicity and some antineoplastic activity [164]. Sorafenib was evaluated in a randomized phase II trial as combined with either temsirolimus (mTOR inhibitor), or tipifarnib (farnesyltransferase inhibitor), demonstrating no sufficient antineoplastic benefit against metastatic melanoma [165]. The combined VEGF/VEGFR blockage was studied in a phase II clinical trial on concomitant use of bevacizumab and sorafenib in advanced malignant melanoma. However, the study demonstrated limited clinical benefit with no reported objective tumor responses and 57% disease stabilization [166]. Importantly, several clinical trials evaluating the use of antiangiogenic drugs for the treatment of CM are currently ongoing or ended with not yet available results [167–178]. A randomized phase II trial testing ipilimumab with or without bevacizumab in patients with unresectable stage III or stage IV melanoma is active [179]. Additionally, a phase I trial is currently ongoing to determine the safety, tolerability and maximum tolerated dosing for the combination of bevacizumab plus ipilimumab in patients with advanced CM [180]. A phase I clinical trial investigating combination of aflibercept and the PD-1 checkpoint inhibitor pembrolizumab in patients with advanced solid tumors including CM, is currently recruiting [181].

Preliminary results from a limited phase II study of sunitinib in patients with CM metastatic to the brain, demonstrated relative disease stabilization and low safety profile [182]. Sunitinib is also among tested drugs in the phase II MATCH screening trial of targeted therapies directed by genetic testing in patients with advanced refractory solid tumors including melanoma, lymphomas, or multiple myeloma [183]. Imatinib has been studied as single agent in several phase II trials for treatment of metastatic CM [184, 185]. An open-labelled, single arm trial of combinational therapy with imatinib and pembrolizumab for unresectable or metastatic KIT-mutant melanoma, refractory to standard therapy, is currently recruiting [186]. In accordance, the combination therapy of imatinib and binimetinib, a MEK inhibitor, is currently under evaluation in a phase II trial in stage III-IV KIT-mutant unresectable CM [187]. The combination of axitinib and nivolumab (anti-PD1) for patients with unresectable stage III or IV CM who have progressed on prior anti-PD1 therapy is currently recruiting [188]. In the same direction, a phase Ib dose-escalation, tolerability and pharmacokinetic study is currently evaluating the humanized anti-PD-1 monoclonal antibody Toripalimab in combination with axitinib in patients with advanced melanoma who failed routine systemic treatment [189]. Cabozantinib, a MET/VEGFR inhibitor, is being tested in combination with immune checkpoint inhibitors in several ongoing phase I/II clinical trials on advanced CM [190–192].

Current clinical trials of antiangiogenic agents in CM are summarized in Table 2.

**Table 2:** Current status of antiangiogenic agents in CM

Drugs affecting angiogenesis	Angiogenic Targets	Main Clinical Studies	Clinical Trial Identifiers
Bevacizumab	VEGF-A	Corrie et al. 2019 [141] Fane et al. 2020 [139]	
Bevacizumab + IFN-α2b	VEGF-A + bFGF	Grignol et al. 2011 [142]	
Nab-paclitaxel + Bevacizumab + Ipilimumab	VEGF-A	Markovic et al. 2020 [140]	
Carboplatin, Paclitaxel, and Bevacizumab ± Everolimus	VEGF-A	McWilliams et al. 2018 [143]	
Ipilimumab + Bevacizumab	VEGF-A		Phase I NCT00790010
Ipilimumab ± Bevacizumab	VEGF-A		Phase II NCT01950390
Pazopanib + Paclitaxel	VEGFR − 1, −2, −3	Fruehauf et al. 2018 [145]	
Aflibercept	VEGF-A, −B, PlGF	Tarhini et al. 2011 [146, 193]	Phase II NCT00450255
Aflibercept ± HD IL-2	VEGF-A, −B, PlGF	Tarhini et al. 2018 [147]	Phase II NCT01258855
Aflibercept + Pembrolizumab	VEGF-A, −B, PlGF		Phase I NCT02298959
Thalidomide	VEGF-A + TNF	Pawlak et al. 2004 [148]	
Thalidomide + Temozolamide	VEGF-A + TNFα	Clark et al. 2010 [149]	Phase II NCT00072163, Phase II NCT00005815, Phase II NCT00104988
Thalidomide + Temozolamide +Lomustine	VEGF-A + TNFα		Phase I NCT00527657, Phase II NCT00072345
Thalidomide + DTIC	VEGF-A + TNFα	Ott et al. 2009 [150]	Phase II NCT00006200
Thalidomide + Semaxanib	VEGF-A + TNFα + VEGFR-2 + KIT		Phase II, NCT00017316
Thalidomide + IFN-α2b	VEGF-A + TNFα + bFGF		Phase II NCT00026520
Thalidomide + PEG-IFN-α2b	VEGF-A + TNFα + bFGF		Phase II NCT00238329
Lenalidomide	VEGF, VEGFR-2	Eisen et al. 2010 [151]	
Lenalidomide + DTIC	VEGF, VEGFR-2	Hwu et al. 2010 [152]	
Imatinib mesylate	PDGFR, KIT	Ugurel et al. 2005 [156] Carvajal et al. 2011 [157]	Phase II NCT00881049, Phase II NCT00154388
Imatinib mesylate + Bevacizumab	VEGF, PDGFR, KIT	Flaherty et al. 2015 [155]	
Imatinib mesylate + Pembrolizumab	VEGF, PDGFR, KIT		Phase II NCT04546074
Imatinib mesylate + Binimetinib	VEGF, PDGFR, KIT		Phase II NCT04598009
Nilotinib	PDGFR, KIT	Guo et al. 2017 [159]	
Axitinib	VEGFR − 1, −2, −3	Fruehauf et al. 2011 [161]	
Axitinib + Paclitaxel/Carboplatin	VEGFR − 1, − 2, − 3	Algazi et al. 2015 [162]	
Axitinib + Nivolumab	VEGFR − 1, − 2, − 3		Phase II NCT04493203
Axitinib + Toripalimab	VEGFR − 1, − 2, − 3		Phase Ib NCT03086174
Endostatin + DTIC	VEGF, VEGFR and bFGF	Cui et al. 2013 [163]	
Sorafenib + DTIC	VEGFR-1–3, PDGFR-β, KIT	Eisen et al. 2011	
Sorafenib + Temsirolimus or Tipifarnib	VEGFR-1–3, PDGFR-β, KIT	Margolin et al. 2012 [165]	
Sorafenib + Lenalidomide	VEGFR-1–3, PDGFR-β, KIT + VEGF + VEGFR-2	Ganesan et al. 2014 [153]	
Sorafenib + Temozolomide	VEGFR-1–3, PDGFR-β, KIT		Phase II NCT00602576
Sorafenib + PEG-IFN-α2b	VEGFR-1–3, PDGFR-β, KIT + VEGF + bFGF		Phase II NCT00623402
Sorafenib + Bevacizumab	VEGF-A + VEGFR-1–3, PDGFR-β, KIT	Mahalingam et al. 2014 [166]	
Ramucirumab ± DTIC	VEGFR-2	Carvajal et al. 2014 [144]	
Bevacizumab + Lenalidomide, Sorafenib,Temsirolimus, 5-fluorouracil, Leucovorin, or Oxaliplatin (FOLFOX)	VEGF + VEGFR-2+ VEGFR-1–3,		Phase I NCT01183663
Sunitinib	PDGFR-β, KIT		Phase II NCT00462982 Phase II NCT02465060 (MATCH TRIAL)
Cabozantenib + Nivolumab/Ipilimumab	VEGFR-2, c-Met, KIT		Phase II NCT04091750
Cabozantenib + Nivolumab	VEGFR-2, c-Met, KIT		Phase I NCT04514484
Cabozantenib + Pembrolizumab	VEGFR-2, c-Met, KIT		Phase II NCT03957551

*Abbreviations*: *bFGF* basic fibroblast growth factor, *TNFα* tumor necrosis factor alpha, *PIGF* placental growth factor, *HD* high dose, *DTIC* Decarbazine, *PEG-interferon* Pegylated Interferon

## Uveal melanoma 

### Epidemiology, diagnosis and therapy

 UM is recognized as a rare cancer; however, it is the second most frequent type of melanoma after its cutaneous counterpart and the most common primary intraocular malignancy [1]. UM represents 85% of all ocular melanoma, whereas remaining cases arise from the conjunctiva (4.8%), and from other sites (10.2%) [194]. The uvea is the vascular layer of the eye, and it is composed of retinal choroid, iris and ciliary body. UM mostly originates from melanocytes located in the choroid (90%), and to a lesser extent in the iris (4%) and ciliary body (6%) [194].

The uveal tract is a capillary-rich tissue lacking of lymphatic vessels; thus, angiogenesis, but not lymphangiogenesis, plays a pivotal role in the UM spreading of metastases [195–197]*.* While CM metastasizes in different organs, UM metastases are confined to a single organ represented by the liver (metastatic hepatotropism in over 90% of cases), thus, it does not follow the usual stepwise progression of the TNM staging system from primary tumor to lymph nodes and then, to distant metastases. Liver metastasization also provides a simpler model for studying and identifying possible treatments [198, 199]*.*

Studies on molecular profiling identified mutations in the GNAQ and GNA11 genes, encoding for Gα subunits of G-proteins, in about 80% of UM patients. This mutation leads to activation of the mitogen-activated protein kinase (MAPK) pathway and oncogenesis in UM [200–202]*.* Other mutations detected in UM include NRAS and NF1 [33, 34].

The main risk factors for UM development include the presence of dysplastic nevus syndrome, choroidal nevi, ocular or oculodermal melanocytosis, familial syndromes including germline BRCA1-associated protein 1 (BAP1) mutations and neurofibromatosis [203–206]. Importantly for this discussion, CM is not a predisposing factor for UM [3, 207].

The incidence of UM has remained relatively unchanged in recent decades, accounting for 5.1 cases per million individuals in the USA and between 1.3–8.6 cases per million in Europe [208–215]. The disease is more common in Caucasians, with a median age of presentation of 60 years and a 30% higher incidence in males [208–210, 212, 216–218]. The diagnosis of UM is primarily clinical; however, fine-needle aspiration biopsy is mandatory for diagnostic accuracy in selected cases [219, 220]. The main determinants of UM metastatic progression include clinical (tumor size, ciliary body involvement, degree of extraocular extension), histopathologic (epithelioid cytomorphology, infiltrating lymphocytes and macrophages, fibrovascular networks) and genetic factors [221–226]. The most frequent mutational event associated with an increased risk of distant recurrences in UM is chromosome 3 complete monosomy. Further cytogenetic aberrations include 8q gain, 1p loss, 6q loss and 8p loss [227, 228].

In recent decades, treatment of primary UM has evolved from enucleation to eye-preserving modalities inclusive of radiation, surgical and laser therapy [229], reaching over 90% local disease control. In addition, advance in UM characterization based on cytogenetics and gene expression profiling has considerably improved early identification of patients at risk of metastatic progression.

However, 50% of patients still develop metastatic disease with reported 15% one-year survival and average life expectancy ranging from 6 to 12 months [1, 194, 211, 230, 231]. There is currently no standard of care for UM in adjuvant and metastatic settings, and available treatments are mostly adapted from CM despite their different clinical and genetic profiles [232].

Unlike the positive results achieved in CM, systemic treatments including immunotherapy and targeted therapies have failed to improve survival outcomes in clinical trials for UM metastatic disease [209, 230, 233–239].

### HIF-regulated pathways

As reported for CM, hypoxia also regulates different pathways involved in the progression of UM, such as metastasization and angiogenesis. Recently, a global and integrated molecular characterization of 80 primary UM led to the discovery of four molecular and clinical types of UM, one of which characterized by distinct features suggestive of hypoxia [240].

#### HIF-1 expression and regulation

While multiple studies reported the role of HIF-1α in UM onset and progression [241–243], only few evidences indicated HIF-2α role in UM [244].

Immunohistochemical analysis of biopsies from UM patients and Gene Set Enrichment Analyses identified a signature containing HIF-1α, among other factors, as the most accurate predictor of UM metastasis [245]. These findings are in agreement with those demonstrating that the expression of HIF-1α was higher in a subtype of UM, i.e. choroidal melanoma, when compared to eyelid nevi and was associated with the tumor size but not with scleral invasion [246].

HIF-1α expression both in cells grown under normoxic conditions and in well-vascularized area demonstrated the relevance of this protein, and more in general of its target genes, also at the beginning of UM growth, when hypoxic regions are not yet evident.

HIF-1α protein stabilization and increased expression of its target genes is evident in UM cells under normoxic condition of growth through a mTOR-dependent mechanism suggesting that mechanisms different from hypoxia could regulate HIF-1α [242, 247–249]*.* In agreement with these results, pharmacological and genetic approaches demonstrated the ability of HIF-1α to promote in vitro invasion of UM cells both in normoxia and hypoxia through a non-canonical Notch pathway involving ERK and AKT [249]. The same group suggested direct pharmacological inhibition of HIF-1α, or indirect through Notch or ERK1/2, also for UM whose microenvironment showed normal oxygen concentration [250, 251].

An increased expression and nuclear localization of HIF-1α was observed after exposure of UM cells to hypoxia, together with HIF-1α-induced VEGF expression [242, 248].

A retrospective analysis performed on UM specimens from about 90 patients evidenced a correlation between the expression of HIF-1α and both proliferative (MIB-1) and vascular (CD31, VEGF-A) markers, as well as necrosis. Since HIF-1α was not restricted to hypoxic regions, as reported above, these results are indicative of an oxygen-independent regulation of HIF-1α expression. The same study did not show any correlation between HIF-1α levels and patient survival. The authors indicate the limited number of patients or the involvement of other factors, such as HIF-2α, as possible cause of the lack of correlation. In addition, differently from what reported for other tumors [235], the authors did not evidence HIF-1α modulation following irradiation [236].

A study carried out by Jager group’s on about 60 patients enucleated for UM and corroborated by data on 80 patients from the Cancer Genome Atlas reported no correlation between HIF-1α expression and basal diameter or tumour thickness, while HIF-1α expression was found positively related with endothelial marker CD31 and with enhanced infiltration of T cells and macrophages. The authors indicated that angiogenesis of UM is related to genetic profile: increased HIF-1α expression and microvascular density were associated with loss of BAP1 expression, a genetic predictor for UM metastatic disease related to microvascular density [9]. As BAP1 loss in UM is related to an enhanced NF-kB expression [237], the authors postulated NF-KB involvement in BAP1 regulation of HIF-1α expression.

In 2012 Giatromanolaki A and collaborators demonstrated upregulation of HIF-1α and HIF-2 α and their target genes, VEGF and LDHA, after exposure of UM cells to hypoxia. More importantly, analysis of about 60 UM treated with enucleation, identified a correlation between HIF-2α, but not HIF-1α, and VEGF expression, VEGFR2/KDR phosphorylation/activation and LDH5 expression. The authors suggested a prominent role of HIF-2α in UM progression through induction of a VEGF/pVEGFR2 autocrine loop and glycolytic enzymes, such as LDH5. The evidence that pVEGFR2 was associated with prognosis supported the relevance of HIF-2α in UM progression, [244]. After this initial paper no other findings indicating the involvement of HIF-2a in UM were reported.

Findings demonstrating HIF-1α regulation by microRNA in UM have been reported by Xia and colleagues, who evidenced promotion of HIF-1α signaling and migration of UM cell by miR-652 through the repression of HOXA9 [252]*,* a negative regulator of HIF-1 expression in cutaneous squamous cell carcinoma [253]*.*

#### Angiogenesis

Similarly to what observed for most solid tumors, angiogenesis plays a pivotal role also in the development and progression of UM [195, 254]. Avascular phase of UM has been suggested to be related to dormancy and to be the cause of slow clinical course of multiple cases of UM, while angiogenic switch can be responsible of a rapid clinical course [255].

With the exception of an old study performed on a small cohort of patients with or without metastases demonstrating that tumour microvessel density was unrelated to UM patient survival [256], several other studies reported tumour vascularity as a marker of aggressiveness in UM patients, and both microvascular density and microvascular patterns (loops and networks) as independent prognostic factors in UM [257–259]. More recently, data from UM patients obtained from The Cancer Genome Atlas evidenced that patients with high “angiogenesis signature” (genes related to hypoxia/vascularization/proangiogenic factors) were those with higher relapse and lower desease-free survival [254].

Exposure of UM cells to hypoxia increased the expression and nuclear localization of HIF-1α and the expression of multiple target genes [242, 248].

Different forms of VEGF, including VEGF-A, along with Angiopoietin-2, MMPs, PDGF, CXCR4, and Met represent some of the proangiogenic factors induced by HIF-1α in UM [242, 247, 260–263]. Exposure of primary short-term UM cultures to hypoxia also led to an increased expression of proinflammatory cytokines such as PLGF, transforming growth factor beta (TGFβ), END1, and ICAM1 and a lower expression of AIMP1, CCL2 (MCP-1), and interleukin-1β, thus indicating the effect of hypoxia on the expression of immune response genes [264]. Other relevant proangiogenic factors found in patients affected by UM include interleukin-6 and -8, RANTES and basic fibroblast growth factor [265–267].

VEGF represents one of the most important angiogenic factors induced by hypoxia in UM. It is expressed by retinal tissue, as well as primary and established UM cells, and its expression is further increased by hypoxic condition [242, 265, 268]. An increased VEGF expression has been also reported when UM tissues were compared to normal ocular ones [263, 265, 269].

High variability of VEGF expression in human UM tissues was observed ranging from 26 to 96% [248, 270, 271]. VEGF expression in UM patients was significantly higher in patients with scleral invasion or with large-sized tumors [263], while other studies reported VEGF association with the tumor size but not scleral invasion [246].

An increased VEGF, as well as HIF-1α expression, was observed after exposure of UM cells to hypoxia [242, 248].

Contrasting results are present in literature evidencing either no correlation between VEGF expressed in UM specimens and the presence of metastatic disease [270], or a positive VEGF correlation with experimental metastases [272–274]. Moreover, while a correlation between VEGF serum levels and metastatic disease has been identified in UM patients identifying VEGF as a biomarker for metastatic UM [262], no association between VEGF-A expression and histopathological characteristics or occurrence of metastasis or metastases-free survival was demonstrated, probably due to the small number of samples or to the short follow-up [242, 261]. From these findings emerged that further studies are necessary to evaluate whether VEGF expressed in the biopsies’ specimens or present in the sera of patients could be considered a marker for following the progression of the disease or for screening purposes.

Surprisingly, the study of Jager MJ group’s performed on patients enucleated for UM, demonstrated that while VEGF-A was not related to microvascular density, VEGF-B negatively correlated with vascularization, thus indicating in UM a previously unknown role of VEGF-B and different from those observed in other types of cancer. The authors also suggested a role of VEGF-A in UM vascularization depending on the status of vasculature [195].

Also, angiopoietin-like 4 represents an important regulator of UM tumor angiogenesis. It is expressed in about 80% of human UM biopsies, is induced by hypoxia and cooperates with VEGF in the promotion of UM angiogenesis [248]. High levels of angiopoietin-like, HIF-1α and VEGF were also observed after intraocular injection of UM cells in mice, as well as in ocular tissues (vitreous samples) from patients with primary UM when compared to healthy people [248, 265, 269, 275]. Inhibition of both angiopoietin-like 4 and VEGF partially reduced angiogenic activity by UM cells, while it was completely blocked by pharmacological inhibition of HIF-1α, thus indicating the relevance of other angiogenic factors, other than VEGF and angiopoietin-4, to promote the angiogenesis of UM [248].

#### Vasculogenic mimicry

Another important mechanism of blood supply in UM is furnished by the vasculogenic mimicry [66, 67]. Starting from the results identifying vasculogenic mimicry in UM and CM [66], several other groups confirmed the role of vasculogenic mimicry in UM [276–279].

VEGF-induced vasculogenic mimicry of UM has been evidenced with the involvement of the PI3k signal pathway [280].

While hypoxia exposure has been reported as a contributor to vasculogenic mimicry in tumors from different origin including CM [281, 282, 283], to the best of our knowledge no data exist about regulation of vasculogenic mimicry by hypoxia in UM.

#### Invasion

Regulation of UM cell adhesion, migration, and invasion by HIF-1α has been reported, with the limit of only one cell line used for the study [243], while in vitro cell proliferation was not affected by HIF-1α [242].

While hypoxia exposure has been reported as a contributor to EMT [281, 282] in tumors from different origin including CM [283], to the best of our knowledge no data exist about regulation of EMT by hypoxia in UM.

Hypoxia also affects UM progression through ECM modulation. A paper published in 2010 demonstrated that simulated hypoxia by cobalt chloride induced the expression of lysyl oxidase, an enzyme belonging to a family of enzymes essential for the assembly of ECM and for the development of normal visual function [284, 285]. The authors found expression of lysyl oxidase in about 80% of primary human UM specimens and association with a more aggressive phenotype and a shorter metastasis-free survival of UM patients. They also demonstrated reduced cell invasion, but no cell proliferation, after inhibition of catalytic activity of the protein and suggested lysyl oxidase as a potential therapeutic target for the therapy of UM [286]. After that paper, no further results regarding involvement of lysyl oxidase in UM have been published.

#### Autophagy

In order to define a possible connection between the expression of hypoxic markers such as HIF-1α/ LDHA and autophagic markers (beclin1, LC3A), in 2011 a paper by Giatromanolaki and colleagues carried out in 99 UM specimens, demonstrated association between autophagy and hypoxia in the perinucler area, where autolysosome are formed. Moreover, overexpression of autophagic markers linked with tumor hypoxia were associated with poor prognosis [287, 288]. These data are in agreement with those reporting hypoxia ability to trigger autophagy [289–291] and are of particular relevance also considering the recent findings demonstrating the role played by autophagy in UM progression [292], as well as, the efficacy of autophagy inhibitors as a possible strategy for the treatment of metastatic UM [293].

### Preclinical studies with drugs targeting HIF-1, angiogenesis or vasculogenic mimicry

#### HIF inhibitors

Identification of drugs targeting HIF-1α could represent a possible strategy for UM therapy, or to eradicate UM cells still present after local radiotherapy [261]. Several in vitro approaches, such as lentivirus-mediated short hairpin RNA delivery or digoxin, a small molecule inhibiting HIF-1, reduced in vitro invasion of UM cells [249]. In order to overcome toxicity and side effects of these approaches, a biocompatible ternary complex was recently developed. It is composed of small interfering RNA against HIF-1α, chitosan, characterized by low toxicity, low immunogenicity, and high biocompatibility [294], and hyaluronic acid to shield the cationic charge of the complexes and facilitate transport in the vitreous humor [295]. This ternary complex demonstrated its efficacy in reducing HIF-1α and VEGF expression under hypoxia, inducing cytotoxicity and reducing migration/invasion of human UM, thus suggesting its possible future application for the local treatment of UM patients. A limit of this study is related to the evidence of efficacy in a single UM cell line and to the lack of in vivo studies [296].

In order to identify new therapy for UM, the group of Halmos G demonstrated the ability of AEZS-108 to induce cytotoxicity in human metastatic UM cells. AEZS-108 is an analog of the Luteinizing Hormone-Releasing Hormone (LHRH) consisting of doxorubicin conjugated to a synthetic peptide containing LHRH that is used against cells expressing the LHRH-receptor. More importantly, the authors identified upregulation of MASPIN/SERPINB5, a tumor suppressor gene for UM [297, 298], as well as downregulation of proteins related to angiogenesis, such as HIF-1α and VEGF, as cause of AEZS-108 efficacy [299]. Given to the pivotal role of angiogenesis in the metastatization of UM [195], the regulation of HIF-1α /VEGF axis by AEZS-108 is of particular relevance. Even if the study presents the limit of only one UM cell lines used, it represents a new opportunity for the treatment of UM positive for LHRH receptor. Previous papers evidenced positivity of LHRH receptor type I in more than 40% of UM specimens [300] and efficacy of LHRH analog to doxorubicin resistant human UM cells, thus indicating the use of AEZS-108 as a possible target therapy for UM treatment [301]. Of note, this drug has already been used in phase I-III clinical trials for the management of endometrial, prostate and ovarian cancer [302, 303].

Recently, systemic administration of the small molecule arylsulfonamide 64B, a chemically stable and lipophilic inhibitor of HIF pathway [304], has been reported to reduce in vivo UM growth and metastasization in orthotopic UM syngeneic and xenogeneic mouse models with minimal toxicity [241]. Affecting the HIF-1/p300/CBP binding, arylsulfonamide 64B also reduced hypoxia-induced expression of CXCR4, involved in UM growth and metastasization [305–307] and c-Met, whose expression is correlated with unfavourable outcome in UM [308, 309]. As SDF-1 and HGF, respectively ligands of CXCR4 and c-Met, are secreted by hepatocytes and could drive the recruitment of circulating UM in the liver, the main site of metastases [310], these results provide the rational for the use of arylsulfonamide 64B for UM treatment.

By using both primary and metastatic cell lines the group of Frenkel demonstrated that UM cell lines are insensitive to hypoxia, and knockdown of CREB through retroviral vectors sensitized UM cells to doxorubicin and DTIC both under normoxia and hypoxia. Silencing of CREB under hypoxia also enhanced apoptosis and reduced the expression of VEGF, thus indicating involvement of CREB in the UM cell resistance to hypoxia [311]. In vivo experiments in mouse models performed by the same group using vectors targeting HIF-1 and CREB blocked tumor growth, thus demonstrating the relevance of the two transcription factors in UM progression and indicated the dependence on glycolysis for the progression of UM. In agreement with these results, the ability of hexokinase 2, the initial and rate-limiting step in glycolysis, to regulate UM growth through the Warburg effect has been described [312]. The relevance of this study is represented by the use of vectors preferentially infecting proliferation cells, such as tumor ones, and by the possibility to use these vectors in combination with chemotherapy with the advantage of eventually reducing chemotherapy concentration and side effects [313].

De Lange and colleagues demonstrated the in vitro and in vivo antitumoral efficacy of restoring p53 activity in UM under either normoxic or hypoxic conditions of growth. In particular, the authors evidenced that Nutlin-3, a Hdm2 antagonist, in combination with RITA, a p53-binding molecule, or with topotecan, a topoisomerase I inhibitor, downregulated HIF-1α expression and reduced in vitro and in vivo growth of UM preclinical models. Superimposable sensitivity of UM cells to the different treatments was observed under normoxic or hypoxic exposure. As p53 mutation is a non-frequent event in UM, combination treatments including p53 activation could represent a possibility of future options for the therapy of UM. Unlikely, no other findings have been reported in the field since the publication of the first study of de Lange [314].

#### Antiangiogenic drugs

Considering the relevance of angiogenesis in the growth and metastases of UM cells and the above reported findings, antiangiogenic therapy represents a potential strategy for UM therapy.

The use of bevacizumab for the treatment of primary or metastatic UM has been investigated in preclinical models, however, contrasting results were obtained. Specifically, the study of Yang and collaborators demonstrated the ability of bevacizumab to reduce in vitro angiogenesis and to suppress hepatic micrometastasis when intravenously injected [315]. Also the findings of Sudaka A et al identified bevacizumab ability to impact in vivo tumor growth as well as to decrease VEGFR2 expression and VEGF content in the tumors, and more importantly, to increase the efficacy of radiotherapy in vitro and in animal models [316]. On the contrary, the study of El Filali group observed an increased growth of intraocularly injected tumors in mice after intraocular injections of bevacizumab [261]. This discrepancy could be due to the different route of bevacizumab administration and consequently to the different concentration of the drug to the eyes, but also to the different models used. The increased tumor growth after bevacizumab treatment could be also related to the induction of HIF-1α and VEGFA, observed when UM cells were exposed in vitro to hypoxia and treated with bevacizumab, thus inducing “pseudohypoxia” as defined by the authors [261, 317]. However, the ability of bevacizumab, and more in general, of antiangiogenic therapy to elicit malignant progression remains an unresolved question. Paradoxical effects, such as enhanced tumour aggressiveness, have been observed also in several tumor histotypes [318–320]. Thus, further studies are necessary before to propose the antiangiogenic therapy for UM treatment.

An in vitro study demonstrated superiority of ranibizumab, a humanized Fab-fragment against VEGF-A, over bevacizumab in suppressing VEGF-A levels and angiogenic potential of UM cells, possibly due to splice variants of VEGF-A or different uptake efficiency and retention of the antibody inside the cells [321].

Contrasting results on antitumor effects also emerged from preclinical studies conducted on imatinib [322–324]. Specifically, imatinib was investigated alone or in combination with the alkylating agent temozolomide. It was demonstrated to antagonize the antiproliferative effects of temozolomide on UM cells in vitro*,* and to improve the antitumor activity of temozolomide in in vivo animal models of UM [322]. Other evidences, however, reported lower proliferation and invasion rates with imatinib treatment when evaluated in animal models of UM, and it was associated with upregulation of the tumor suppressor genes KISS1 and Maspin [324].

The combination of lenalidomide and sorafenib resulted in synergistic inhibition of human UM cells ability to migrate and form tubes, and of tumors growth and metastasis development in a xenograft model [325].

Antitumoral activity in in vitro and in vivo UM models through reduction of NF-KB-mediated VEGF-C secretion and consequently angiogenesis has been observed after inhibition of neddylation, a pathway regulating the turnover of proteins involved in oncogenic transformation and pathogenesis. The authors also evidenced VEGF-C as a mediator of the angiogenesis in the liver, the main organ of UM metastasization. Inhibition of neddylation in UM cells expressing components of neddylation machinery, such as NEDD8-activating enzyme (NAE), also impaired maintenance of cancer stem cells and, more importantly, inhibited liver metastasis in UM cells xenografted in immunodepressed mouse models [326]. Inhibition of neddylation also reduced the expression of the Bcl-xL, a poor prognostic factor for UM, whose inhibition has been reported to induce antitumoral activity in UM [327]. These findings indicate the neddylation pathway as a potential target for the treatment of UM patients carrying liver metastasis either alone or in combination with Bcl-xL inhibitors. The relevance of VEGF-C in UM is also supported by its secretion by UM cells [328].

Preclinical studies also identified the possibility to reduce vasculogenic mimicry in UM as a strategy to inhibit its progression. In particular, in vitro and in vivo experiments carried out by injecting UM cells into nude mice demonstrated the ability of genistein, a natural compound belonging to the class of isoflavones, to reduce vasculogenic mimicry through down-regulation of VE-cadherin [278]*.* The same group also reported the relevance of genistein to block HIF-1α/VEGF axis in non-malignant ocular disease and its relevance in inhibiting ocular neovascularization [329–332], thus supporting the need of further study for its use for the treatment of UM.

Inhibition of vasculogenic mimicry has been also observed when UM cells cultured on a three-dimensional matrix were treated with a chemically modified tetracycline (CMT-3, COL-3), an inhibitor of MMP2, MMP9 and MT1-MMP [279]. Even if contrasting results are reported about the side effects of the tetracycline used in the study, further studies may shed light on the possibility to use this drug to target tumor microenvironment of UM [333, 334]*.*

Inhibition of vascular mimicry, as well as endothelium-dependent vessels and factors such as EphA2, PI3K, MMP2, and MMP9 was also observed after treatment with curcumin in a murine choroidal melanoma model [335]*.*

Table 3 reports a list of preclinical studies with HIF inhibitors and antiangiogenic drugs in UM.

**Table 3:** Preclinical studies with drugs targeting HIF-1, angiogenesis or vasculogenic mimicry in Uveal Melanoma

Drug	Involved Pathways	References
Chitosan	Induced cytotoxicity and reduced migration/invasion; reduced HIF-1α and VEGF expression	Xie L et al Int J Pharm 2020 [296]
AEZS-108	Upregulation of MASPIN/SERPINB5; downregulation HIF-1α and VEGF	Fodor K et al Oncotarget 2020 [299]
Arylsulfonamide 64B	Reduced hypoxia-induced expression of CXCR4 and c-Met, targeting HIF-1/p300/CBP binding	Dong L et al Clin Cancer Res 2019 [241]
Nutlin-3 in combination with RITA or Topotecan	Reduced in vitro and in vivo growth through downregulation of HIF-1α expression	De Lange J et al Oncogene 2012 [314]
Bevacizumab	Reduced in vitro angiogenesis and hepatic micrometastasis; decreased in vivo tumor growth, increased efficacy of radiotherapy in vitro and in vivo; decreased VEGFR2 and VEGF; increased growth of intraocularly injected tumors in mice	Yang H et al Investig Ophthalmol. Vis Sci 2010 [315]; Sudaka A et al Invest New Drugs. 2013 [316]; El Filali M et al Dev Ophthalmol 2012 [261]
Ranibizumab, humanized Fab-fragment against VEGF-A	Angiogenic potential of uveal melanoma cells, suppression of VEGF-A levels	Tura A et al Cancers 2019 [321]
Imatinib	Antagonization of temozolomide antiproliferative effect in vitro and improvement of the antitumor activity of temozolomide in vivo; reduced proliferation and invasion and upregulation of the tumor suppressor genes KISS1 and Maspin	Triozzi PL et al Melanoma Res 2008 [322]; Fernandes BF et al Anal Cell Pathol 2011 [324]
Lenalidomide and Sorafenib in combination	Synergistic inhibition of migration and formation of tubes in vitro, tumors growth and metastasis development in vivo	Mangiameli DP et al J Transl Med 2007 [325]
MLN4924 (Neddylation inhibition)	Reduced angiogenesis through NF-KB-mediated VEGFC secretion; NEDD8-activating enzyme inhibition; reduced expression of the Bcl-xL protein	Jin Y et al Clin Cancer Res 2018 [326]; Némati F et al PLoS One 2014 [327]
Genistein	Reduced vasculogenic mimicry through down-regulation of VE-cadherin; inhibition of ocular neovascularization blocking HIF-1α/VEGF axis	Cong R et al J Exp Clin Cancer Res 2009 [278]; Wang B et al Methods Find Exp Clin Pharmacol; Wang B et al J Ocul Pharmacol Ther 2005 [330]; Wang B et al J Ocul Pharmacol Ther 2003 [331]; Pan JS et al J Ocul Pharmacol Ther 2006 [332].
CMT-3, COL-3	Inhibition of vasculogenic mimicry through repression of MMP2, MMP9 and MT1-MMP	Seftor REB et al AACR 2002 [279]
Curcumin	Inhibition of vascular mimicry, endothelium-dependent vessels, EphA2, PI3K, MMP-2, and MMP-9 expression	Chen LX et al Cancer Biol Ther 2011 [335]

### Clinical trials with antiangiogenic drugs

Starting from 2007, several pilot or phase II clinical trials have been carried out with several antiangiogenic agents, including inhibitors of VEGFR − 1, − 2, and − 3, PDGFR-α and -β, for the treatment of metastatic UM, but disappointing results were obtained, probably due to the small cohort of the recruited patients invalidating the reliability of results [146, 161, 336–346]. No objective responses were observed in most studies [193, 337–340, 342, 343]. Importantly, stabilization of the disease was achieved in more than 50% of patients with some antiangiogenetic agents [146, 336, 341, 344, 347]. However, this information should be taken with caution as most studies were not placebo-controlled [163, 281, 286]. Adverse events were mild in most studies, and reversible with adjustment of dose and/or drug-free period [336, 341, 342, 344]. Furthermore, survival outcomes with antiangiogenic drugs were superior in the UM cohorts compared to CM, probably related to the high vascular density of the uveal tract making it more prone to metastasize directly via the hematogenous route [193, 347].

The STREAM study, the largest trial performed thus far on the use of antiangiogenic drugs for the treatment of metastatic UM, evaluated the efficacy of sorafenib in 147 chemonaïve patients. In this randomized, controlled, phase II study, all patients were initially treated with sorafenib for 56 days in a run-in period. After that, patients with stable disease were randomly assigned to blinded sorafenib or placebo, and further monitored. The trial demonstrated the best observed response rate of 1.7% among all available trials, and a significant increase in mPFS in patients that continued on sorafenib in comparison with placebo. The mOS of the sorafenib group was not different from the placebo group, and compared favourably with previous findings on the use of antiangiogenics in patients with metastatic UM [342–344, 348]. However, cross-over to sorafenib was allowed following progression to placebo, thus questioning the reliability of results [344].

The group of Daud et al. performed a phase II randomised discontinuation trial investigating cabozantinib in patients with metastatic melanoma, including UM, and receiving no more than two prior systemic treatments. All participants were treated with cabozantinib, and then randomised to receive cabozantinib or placebo in case of disease stability. This study suggested potential for clinical activity of cabozantinib in metastatic UM [343]. Recently, a randomized phase II trial evaluated cabozantinib chemotherapy versus temozolomide or DTIC in metastatic UM (Alliance A091201). Unfortunately, treatment with cabozantinib did not improve mPFS, and it resulted in increased toxicity in metastatic UM [349].

In light of all these findings, available results on the use of antiangiogenic drugs for the treatment of metastatic UM indicated some clinical benefit, but the limited study populations prevented any conclusion from being drawn. Thus, participation of patients in clinical trials needs to be encouraged, and further insight is mandatory in order to improve outcomes with antiangiogenic therapies in UM.

In this direction, a number of phase I/phase II clinical trials investigating antiangiogenic therapies in UM are currently ongoing or ended but with not yet available published results [171, 173, 177, 350–354].

The antiangiogenic activity of interleukin-12 was investigated in a randomized phase II trial of a vaccine combining tyrosinase/gp100 peptides emulsified with montanide ISA 51 for stages III and IV melanoma, inclusive of UM [355]. Similarly, a phase II randomized trial of a vaccine therapy and interleukin-12 with either alum or granulocyte-macrophage colony-stimulating factor in treating patients having undergone surgery for stage II, stage III, or stage IV skin or ocular melanoma has been performed [356].

Furthermore, antiangiogenic drugs have been also successfully used for the treatment of side effects after brachy- or radio-therapy for UM [381–384]

A phase II/III clinical trial is currently investigating the use of intravitreal ranibizumab or triamcinolone acetonide compared to no treatment in patients with radiation retinopathy [357, 385]. Interestingly, a randomized phase II trial is currently evaluating the effects of intravitreal aflibercept after stereotactic radiotherapy for the prevention of radiation maculopathy and to promote local disease control in patients with UM [358] Moreover, intravitreal aflibercept is being tested in a randomized phase III clinical trial for prevention of neovascular glaucoma after proton therapy for choroidal melanoma [359].

The clinical trials currently evaluating antiangiogenic agents in UM are listed in Table 4.

**Table 4:** Current status of antiangiogenic agents in UM

Drugs affecting angiogenesis	Angiogenic Targets	Main Clinical Studies	Clinical Trial Identifiers
Bevacizumab + Temozolomide	VEGF-A	Piperno-Neumann et al. 2013, 2016 [342, 345]	
Bevacizumab + IFN-α2b	VEGF-A + bFGF	Guenterberg et al. 2011 [336]	
Thalidomide + IFN-α2b	VEGF-A + TNFα + bFGF	Solti et al. 2007 [337]	
Thalidomide + PEG-IFN-α2b	VEGF-A + TNFα + bFGF		Phase II, NCT00238329
Temozolamide + Thalidomide	VEGF-A + TNFα		Phase I/II, NCT00005815
Temozolomide + Thalidomide + Lomustine	VEGF-A + TNFα		Phase II, NCT00072345
Temozolomide + Sunitinib	VEGFR-1–3, PDGFR, KIT		Phase II, NCT01005472
Aflibercept	VEGF-A, −B, PIGF	Tarhini et al. 2011 [146, 193]	
Lenalidomide	VEGF, VEGFR-2	Zeldis et al. 2009 [339]	
Carboplatin + Paclitaxel + Sorafenib	VEGFR-1–3, PDGFR-β, KIT	Bhatia et al. 2012 [340, 348]	
Sorafenib	VEGFR-1–3, PDGFR-β, KIT	Mouriaux et al. 2016 [346] Scheulen et al. 2017 [344]	
Sunitinib	VEGFR-1–3, PDGFR, KIT	Mahipal et al., 2012 [341]	
Sunitinib vs DTIC	VEGFR-1–3, PDGFR, KIT		Phase II, NCT01551459
Sunitinib vs Valproic Acid	VEGFR-1–3, PDGFR, KIT		Phase II, NCT02068586
Sunitinib + Tamoxifen + Cisplatin	VEGFR-1–3, PDGFR, KIT		Phase II, NCT00489944
Imatinib mesylate	PDGFR, KIT	Hofmann et al. 2009 [338]	
Axitinib	VEGFR − 1, −2, −3	Fruehauf et al. 2011 [161]	
Cabozantinib	VEGFR-2, c-Met, KIT	Daud et al. 2017 [343, 347]	
Cabozantinib vs Temozolomide or DTIC	VEGFR-2, c-Met, KIT	Luke et al. 2020 [349]	
Vaccine: Tyrosinase/gp100 ± Interleukin-12	bFGF, VEGFR-3		Phase II, NCT00003339
Vaccine:Tyrosinase/GP100/MART-1 + Interleukin-12 + Alum/GM-CSF	bFGF, VEGFR-3		Phase II, NCT00031733
Nab-paclitaxel + Bevacizumab vs Ipilimumab	VEGF-A		Phase II, NCT02158520

*Abbreviations*: *NAE* NEDD8-activating enzyme NAE, *bFGF* basic fibroblast growth factor, *TNFα* tumor necrosis factor alpha, *PIGF* placental growth factor, *PEG-interferon* Pegylated Interferon, *DTIC* Decarbazine, *GM-CSF* granulocyte-macrophage colony-stimulating factor

## Mucosal melanoma 

MM is a rare but aggressive tumor, mostly arising from melanocytes located in the mucosal membranes lining the respiratory, gastrointestinal and genitourinary tract [1, 360–362]. MM comprise 0.03% of all cancer diagnoses and 1.3% of all reported melanoma, with the most prevalent site of origin represented by the head and neck region (55.4%), followed by anal/rectal area (23.8%), and the vulvovaginal region (18%) [1, 360–363].

Unlike CM, exposure to ultraviolet light does not appear to be a predisposing factor in the development of MM [360, 362].

The most frequent driver mutated gene in MM is KIT [364]. Specifically, KIT mutation has been identified in over 20% of all MM [360, 362, 365]. However, recent genetic studies and molecular profiling indicated the presence of BRAF mutation in approximately 30% of conjunctival melanoma. Of note, conjunctival melanoma is a subtype of MM that originates from melanocytes in the basal layer of the conjunctival epithelium. However, it is genetically similar to CM based on associated BRAF and NRAS mutations, thus therapies effective at treating CM appear to have clinical benefits in conjunctival melanoma [366]. BRAF mutation is not frequently observed in other types of MM [33], thus conjunctival melanoma representing a molecularly distinct MM subtype. Other mutations detected in MM include NRAS and NF1 [33, 34].

Contrary to the increasing incidence of CM, the incidence of MM has remained stable over the last few decades [360, 367]. Both lymphatic and blood vessels represent the routes of MM dissemination [1, 363, 366]. Due to its rarity, there is currently no universal TNM staging system for MM, and the TNM classification is currently used only for head and neck MM [360, 362, 363].

MM presents worse prognosis compared to CM, with an estimated overall five-year survival rate of approximately 25.0% [1]. This could be possibly related to a late diagnosis due to anatomic location, or to the rich lymphatic and vascular supply of the mucosa [360, 362]. In accordance, the presence of positive lymph nodes has been associated with a 5-year survival of 16.4% compared with 38.7% for patients with negative lymph nodes, thus indicating the important prognostic effect of lymph node status in MM [1].

An intense expression of VEGF and VEGFR-2 in oral MM cells was demonstrated in their early invasive phase, suggesting the role of VEGF/VEGFR-2 axis in disease progression [368]. Also, the prognostic significance of vasculogenic mimicry on oral MM was evaluated in in vitro studies. In detail, the presence of PAS-positive loops, indicative of vasculogenic mimicry and networks, was a strong independent prognostic factor of OS, and it was also associated with lymphogenous and hematogenous metastasis [369]. To the best of our knowledge no findings were reported about the role of hypoxia in MM progression.

The treatment of choice of primary MM is represented by wide excision surgery. Radiation therapy allows for local disease control when resection is not feasible, or may be used adjuvantly to enhance locoregional control, but the effect on survival remains questionable given the limited literature evidence [360, 362, 363].

To date, there are no prospective randomized studies or consensus guidelines to establish the optimal systemic therapy for metastatic MM [370].

Clinical activity for KIT positive metastatic MM has been observed in prospective trials with KIT inhibitors exhibiting antiangiogenic properties [157, 159, 371–374]. Specifically, the use of sunitinib for patients with metastatic MM was investigated in a multicentre phase II study, showing limited activity, poor tolerability and no prolonged responses [371]. Similarly, the efficacy of dasatinib, a protein tyrosine kinase inhibitor, was studied in the context of a phase II trial on advanced MM demonstrating poor response rates [372].

Results from phase II trials on nilotinib, a KIT-selective tyrosine kinase inhibitor, demonstrated some clinical benefit in KIT positive metastatic MM with or without prior progression on KIT inhibitor treatment [159, 374]. However, the therapy of choice for patients with KIT mutation is still represented by imatinib based on significant activity resulting from phase II clinical trials [157, 373].

In recent years, improved survival outcomes in MM have been reported with immunotherapy [367, 375–378]. Some clinical trials are now focusing on the use of immune checkpoint inhibitors in combination with antiangiogenic agents in metastatic MM. A multicenter, single-arm, open, phase II clinical trial evaluating the efficacy and safety of toripalimab, a PD-1 checkpoint inhibitor, after chemotherapy in combination with endostatin in patients with unresectable locally advanced or metastatic MM, is ongoing [379]. Also a combination phase II trial of axitinib and nivolumab for patients with unresectable or advanced MM who have progressed on prior anti-PD1 therapy, is currently recruiting [188]. Similarly, a phase II study is currently investigating bevacizumab in combination with the anti-PD-L1 checkpoint inhibitor atezolizumab in patients with unresectable locally advanced or metastatic MM [380].

## Conclusions

The oxygenation represents a crucial factor of the tumors microenvironment which could affect the behavior of both tumors and stromal cells through the regulation of genes involved in apoptosis, cell survival, glucose metabolism, angiogenesis, as well as response to therapy.

Hypoxia plays an essential role in the development and progression of CM and UM, regulating the main signalling pathways involved in tumour progression and resistance to therapies. In melanocytes and melanoma, in addition to hypoxia, HIF-1 is also regulated in an oxygen-independent manner, thus indicating that melanoma pathogenesis is determined by a balance between oxygen-dependent and oxygen-independent mechanisms.

The definition of molecular pathways involved in melanoma pathobiology is an urgent need that could lead to the identification of new agents for prospective clinical trials. Drugs targeting HIF-1, angiogenesis or vasculogenic mimicry have demonstrated promising results in preclinical studies, especially for those related to CM and UM. The efficacy of antiangiogenic drugs has been evaluated in the context of clinical trials in all metastatic melanoma subtypes, or in patients at high risk of disease progression, as single agent or in combination with other strategies, reporting some improvement in survival outcomes.

Importantly, based on close interaction between angiogenesis and the immune microenvironment, several studies evaluated the combination of antiangiogenic therapies and checkpoint inhibitors in all melanoma subtypes. However, the evidences supporting the use of antiangiogenic therapies in metastatic melanoma are still insufficient, and no effective strategy targeting vasculogenic mimicry has been developed so far. Therefore, further studies are warranted to expand the assessment of antiangiogenic and antivascular mimicry agents and molecules targeting HIF alone or in combination therapies, and continued participation in clinical trials should be encouraged. This could hopefully bring to the identification of new therapeutic options, and to increase survival benefit of melanoma patients with recurrent or metastatic disease.

## Data Availability

Not applicable.

## Abbreviations

CM: Cutaneous melanoma
UM: Uveal melanoma
MM: Mucosal melanoma
EMT: Epithelial-mesenchymal transition
HIF: Hypoxia-inducible factor
HRE: Hypoxia-responsive elements
PD-1: Programmed death receptor-1
CTLA-4: T-lymphocyte–associated antigen 4
VEGF: Vascular endothelial growth factor
PDGFR: Platelet-derived growth factor receptor
MITF: Microphthalmia-associated transcription factor
MMP: Matrix metalloproteinases
ET-1: Endothelin-1
ECM: Extracellular matrix
uPAR: Urokinase-type plasminogen activator receptor
FAK: Focal adhesion kinase
GLUT1/4: Glucose transporters 1/4
LDHA: Lactate dehydrogenase A
LC3A: Light chain 3 A
HGF: Hepatocyte growth factor
mAb: Monoclonal antibody
RECIST: Response Evaluation Criteria in Solid Tumors
mPFS: Recorded median progression-free survival
BAP1: BRCA1-associated protein 1
LHRH: Luteinizing Hormone-Releasing Hormone
mOS: Median of overall survival
VEGF-R: Vascular endothelial growth factor receptor
TGFβ: Transforming growth factor beta
PDGF: Platelet-derived growth factor
EGF-R: Epidermal growth factor receptor
